# Changes in Electroencephalography Activity of Sensory Areas Linked to Car Sickness in Real Driving Conditions

**DOI:** 10.3389/fnhum.2021.809714

**Published:** 2022-02-08

**Authors:** Eléonore H. Henry, Clément Bougard, Christophe Bourdin, Lionel Bringoux

**Affiliations:** ^1^Stellantis, Centre Technique de Vélizy, Vélizy-Villacoublay, France; ^2^Aix Marseille Univ, CNRS, ISM, Marseille, France

**Keywords:** EEG activity, car sickness, real driving, car passenger, sensory integration

## Abstract

Car sickness is a major concern for car passengers, and with the development of autonomous vehicles, increasing numbers of car occupants are likely to be affected. Previous laboratory studies have used EEG measurements to better understand the cerebral changes linked to symptoms. However, the dynamics of motion in labs/simulators differ from those of a real car. This study sought to identify specific cerebral changes associated with the level of car sickness experienced in real driving conditions. Nine healthy volunteers participated as front passengers in a slalom session inducing lateral movements at very low frequency (0.2 Hz). They were continuously monitored via EEG recordings and subjectively rated their level of symptoms after each slalom, using a 5-point likert scale. Car-sickness symptoms evolved concomitantly with changes in theta and alpha power in the occipital and parietal areas. These changes may reflect altered sensory integration, as well as a possible influence of sleepiness mitigating symptoms.

## Introduction

Motion sickness is common when the body is exposed to particular kinds of movement during passive locomotion in vehicles, especially in land vehicles (Reason and Brand, 1975; Lacount et al., 2009; Green, 2016). A physiological response to such movement gradually induces evolving symptoms, from mild stomach aches or headaches to dizziness, nausea, and eventually vomiting (Green, 2016; Dennison, 2017). Motion sickness is thus considered as a neuro-vegetative crisis that can potentially lead to significant physical disorders, depending on individual susceptibility (Duclay, 2008; Murdin et al., 2011).

Cars, the most common form of land transportation, induce a specific form of motion sickness referred to as car sickness (Murdin et al., 2011), where passengers are the most likely to develop symptoms (Diels and Bos, 2015). The current development of autonomous vehicles, whose drivers will become passengers in their own vehicle, may well increase the number of car occupants affected. Although the causes of car sickness have yet to be fully identified, several hypotheses related to movement perception (acceleration, frequency, duration, axis) have been proposed. Very low-frequency movements were found to induce motion sickness, especially oscillating movements between 0.10 and 0.50 (Hz) (Turner and Griffin, 1999; Nakashima and Cheung, 2006). Pioneer work under laboratory conditions explored motion sickness occurring in the vertical axis (O’Hanlon and McCauley, 1974). When these data were modelized, a critical threshold between 0.16 and 0.20 Hz was identified as inducing the highest level of motion sickness incidence. Furthermore, it was shown that the greater the acceleration, the quicker and more severe the symptoms (O’Hanlon and McCauley, 1974; Bos and Bles, 1998). More recently, various attempts have been made to identify the dynamic constraints specifically affecting car passenger comfort, i.e., movements perceived in the longitudinal and lateral axis (Nakashima and Cheung, 2006). Several authors analyzed the impact of very low-frequency lateral movements (0.16–0.20 Hz) in real driving conditions using reproducible slaloms (Wada et al., 2012; Wada and Yoshida, 2016; Kuiper et al., 2018). All the studies confirmed that these lateral movements induce car sickness. This particular frequency of movements (0.20 Hz) appears to be noxious because it is perceived as a zone of uncertainty in the reference frame that allows the position and displacement of the body in space to be established (Lichtenberg et al., 1982; Merfeld et al., 1999). More precisely, inertial forces tend to be interpreted as translational above 0.20 Hz, while below this value they tend to be interpreted as tilt by the vestibular system (Bos and Bles, 1998). These observations are in line with the otolith Tilt-Translation segregation hypothesis suggesting that head stimulation at this frequency particularly yields ambiguous otolith information about type of motion (tilt vs. translation; Wood, 2002).

Ambiguous sensory information is thus commonly considered a major cause of motion sickness. One leading theory postulates that motion sickness occurs when the central nervous system receives conflicting multi-sensory information about movements (Reason and Brand, 1975), particularly from the visual, vestibular, and proprioceptive systems (Schmäl, 2013). For example, when traveling in a car with limited outward visibility, the visual system suggests the body is not moving, whereas inputs from the vestibular and proprioceptive systems report motion (Murdin et al., 2011; Bronstein et al., 2013). The multimodal integration of these sensory inputs at cerebral level therefore appears to play a large role in the onset of motion sickness (Chen et al., 2010; Schmäl, 2013). The occipital area is known to be responsible for visual input integration, while the parietal and central areas are involved in proprioceptive and vestibular input integration. It is the combination of and coordination between these different areas that allow precise and robust perception of the individual’s motion relative to his/her environment (Schmäl, 2013). Of the many methods of monitoring cerebral activity, electroencephalography (EEG) is one of the most widely used because of its high temporal resolution and portability (Chen et al., 2010; Naqvi et al., 2015). This type of analysis is used to characterize cerebral activity according to time/frequency domain. For example, the power in different frequency bands indicates the level of activation of a given area. More precisely, low frequency bands such as delta (0.1–3 Hz), theta (4–7 Hz), and alpha (8–12 Hz) are mainly considered as markers of slow cerebral activity, while beta (13–20 Hz), a medium frequency band, indicates a normal level of cerebral activation (Picot et al., 2009; Sauvet et al., 2014).

Cerebral changes induced by motion sickness were addressed in various studies using EEG measurements. Nonetheless, the motion sickness in these experiments was induced by different means, like parallel swing, drum rotation, Visually Induced Motion Sickness (VIMS) (3D videos, virtual reality, static driving simulator) (Koohestani et al., 2019), leading to inconsistent results. In parallel swing and drum rotation studies, the main motion-sickness-induced modifications in EEG activity were observed in the frontal and central area, specifically in the theta band (Wu, 1992; Wood et al., 1994). Studies conducted with VIMS showed changes in frontal and temporal areas, especially in the theta and beta bands (Naqvi et al., 2015; Liu et al., 2017). Some studies specifically addressing car sickness used a static driving simulator (Min et al., 2004; Park et al., 2008) and reported changes in frontal, central and occipital areas in several frequency bands (delta, theta, alpha and beta). However, as it involves no physical movement, this static simulator set-up can be considered a VIMS device. In order to create a more realistic and controlled driving environment, others used a dynamic driving simulator (combination of virtual reality + physical movements) (Lin et al., 2007, 2013; Chen et al., 2010; Chuang et al., 2016). They all reported changes in occipital, parietal and somatosensory areas, mainly in the delta, alpha, and theta bands (Chen et al., 2010). While the use of a dynamic driving simulator offers the advantage of soliciting multimodal sensory inputs (vestibular, proprioceptive, and visual), however, such an experimental paradigm may not distinguish cerebral changes linked to car-sickness symptoms from those induced by virtual reality (VR).

Currently, it is widely accepted that the dynamics of motion used in laboratory conditions (drum rotation, VIMS, dynamic driving simulator) are quite different from those in a real car. Here, we sought to avoid this bias and to identify specific cerebral changes associated with car sickness by conducting our experiment in real driving conditions, using very low-frequency lateral movements and synchronized EEG recordings. Based on previous findings, we hypothesized that changes in cerebral activity would occur concomitantly with car sickness, specifically in cerebral areas allowing the multimodal integration of visual, vestibular, and proprioceptive inputs.

## Materials and Methods

### Participants

Nine healthy participants (5 women and 4 men, age (mean ± SD): 38.8 ± 7.5 years) volunteered to take part in this study. To be included in the study, participants had to be 18 years old minimum and must hold a driver’s license for at least 2 years. They did not report any vestibular or neurological disorders and had not drunk any alcoholic beverages for 24 h before the experiment. To guarantee sample homogeneity, the participants were selected according to their answers to the Motion Sickness Susceptibility Questionnaire (MSSQ; Golding, 2006). The mean percentile score was 89.8 ± 9.9%, indicating high susceptibility to motion and car sickness. After being informed of the various procedures and general objectives of the study, all volunteers signed a consent form. The participants were warned that they might become motion sick during the experimental sessions and told they could stop the experiment at any time and for any reason. They were not paid for their participation and no conflict of interest was declared. This study was approved by the local ethics committee of Aix-Marseille University in accordance with the ethical standards laid down in the 1964 Declaration of Helsinki.

### Experimental Set-Up

**FIGURE 1:**
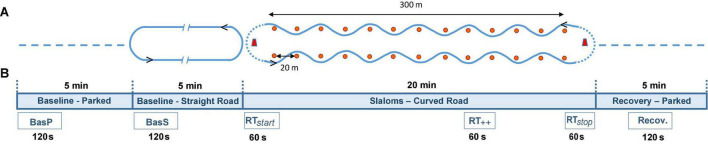
Representation of **(A)** test set-up and timeline of the test session with each period: two baseline periods (parked and straight road), slaloms and recovery period; **(B)** the six time intervals per session analyzed for EEG signal recordings: baseline parked (BasP), baseline straight road (BasS), slaloms (comprising RT_start_, RT_++_, RT_stop_) and recovery (Recov); see section “Data Acquisition and Processing”.

Tests took place in a private area approximately 400 m long and 50 m wide, with no other traffic present, for controllability and safety reasons. The vehicle used for these tests was a medium-sized car popular in France (Citroën C4 Picasso), driven by one of two professional drivers specifically trained to run these sessions with the highest reproducibility. Participants were seated in the front passenger seat of the vehicle in a predefined sitting position and with their safety belts fastened. They were equipped with an EEG device to measure their cerebral activity and a slider was positioned in front of them to allow them to rate their car-sickness level (equipment detailed below). The EEG headset, the car-sickness rating slider, and the controller area network (CAN) data from the vehicle were connected to a laptop in the rear seat of the vehicle for synchronization. The experimental road was an oval track consisting of two straight segments approximately 300 m long with turning areas of 10 m radii at each end. Twelve pylons were located 20 m apart along both straight segments (Figure 1A).

### Procedure

Every test session began with two baseline periods (one in static conditions, parked for 5 min, called baseline parked); one in dynamic conditions (straight road with U-turns for 5 min, called baseline straight road) during which participants had to keep their eyes open for 2 min, close their eyes for 1 min, and again keep them open for 2 min. Signal quality assessment was performed visually (online checking) during the 2 baselines—(parked and straight road). Next came a slalom period of about 20 min to induce carsickness symptoms (shorter if participants felt too sick to finish the test, i.e., maximum score for discomfort (4) on the subjective scale). Finally, after stopping the vehicle, there was a static recovery period (5 min) with eyes open. During the baseline periods (baseline parked and baseline straight road) and the parked recovery period, participants were instructed not to move (head, trunk); during the slalom period, they were instructed to look forward and ahead and to move as little as possible. On completion of the test, participants were debriefed and released. Each test session lasted approximately 1 h (participant equipment, tests and debriefing), divided into periods used for data analysis: Baseline parked, baseline straight road, slaloms and recovery (Figure 1A). During the baseline straight road and the slalom periods, the driver drove at a continuous speed of approximately 35 km/h, and at approximately 15 km/h where U-turns were required, so as to minimize lateral accelerations. In the slalom period, the driver executed zigzags to the left and right of the pylons to induce reproducible lateral acceleration levels. The gap between pylons and the car speed ensured lateral movements of close to 0.2 Hz, recognized as a car- sickness-inducing stimulus (Bos and Bles, 1998). The mean time for one slalom and mean time for the whole slalom period were 30 ± 3 s; 19 ± 1 min, respectively. The mean ± SEM of the resulting lateral oscillation frequencies and accelerations in the ecological conditions were 0.20 ± 0.003 Hz and 2.03 ± 0.04 m.s^2^, respectively.

### Data Acquisition and Processing

Due to technical constraints from EEG signal processing (minimum recording length of 60 s), six different phases of recordings were selected for further analyses [(1) baseline parked (BasP—first 120 s), (2) baseline straight road (BasS—first 120 s), (3) the first round trip (RTstart–60 s), (4) the highest-rated round trip (RT + + –60 s), (5) the last round trip (RTstop–60 s), (6) recovery (Recov—the middle 120 s) (Figure 1B)]. The total amount of extracted recordings thus corresponded to 9 min for each participant. Each round trip was considered as two consecutive slaloms (back and forth, excluding the U-turns, as the latter events did not respect the car dynamics investigated in the study). Reference measurements were obtained from the baseline parked period. To link car-sickness ratings with EEG recordings, ratings obtained from each slalom were considered in pairs to calculate an average rating for the round trip (back and forth, mean of two successive scores). For each participant, RT_++_ was identified using the highest MS level, as suggested by Chen et al. (2010), and was averaged with the rating of the following slalom. Finally, subsequent reactions were assessed via a rating recorded after 2 min of recovery.

### Car-Sickness Ratings

The test included regular subjective assessments of car-sickness severity and evolution, conducted to analyze car-sickness symptoms from their very first appearance. A 5-point likert scale was used, representing the first five levels of Griffin and Newman’s scale graduated from 0 to 4: 0 = No symptom, 1 = Any symptom, however slight, 2 = Mild symptoms, for example, stomach awareness but no nausea, 3 = Mild nausea, 4 = Mild to moderate nausea (as used in recent studies by Green, 2016; Wada and Yoshida, 2016). Participants were instructed to indicate their discomfort level using the slider (Griffin and Newman, 2004; Wada et al., 2012). Only one score was recorded for baseline parked and one for the recovery period. For the slalom period, participants were asked to record their rating based on their worst experience of symptoms during the U-turns at the end of the slalom. One advantage of this method is that car-sickness symptoms are rated promptly, without any memory effect.

### Electroencephalography Recordings

**FIGURE 2:**
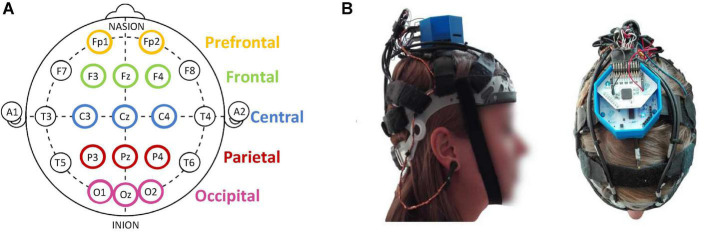
Illustration of **(A)** electrode positioning based on a modified International 10–20 Electrode Placement System; **(B)** EEG headset (14 dry and active Conscious Labs electrodes (https://conscious-labs.com)) with the OpenBCI (http://openbci.com) acquisition card (right).

Participants were continuously monitored for EEG recordings with 14 dry active flexible polymer electrodes mounted on finely adjustable straps, so as to minimize discomfort and not influence car-sickness symptoms (Conscious Labs). EEG signals were recorded using opensource acquisition software OpenBCI GUI (OpenBCI). Electrodes were placed according to a modified International 10–20 Electrode Placement System (Chuang et al., 2016) with Fp1, Fp2, F4, Fz, F3, C4, Cz, C3, P4, Pz, P3, O1, Oz, and O2 (Figure 2). In addition to these acquisition electrodes, an ear-clip electrode was used for bias and another for reference, each positioned at the earlobes. The sampling rate of the EEG data was 125 Hz. The headsets for the EEG recordings were chosen on the basis of their ready availability and of the high quality of their EEG signals, which enabled impedance and movement artifacts to be reduced.

### Electroencephalography Data Analysis

**FIGURE 3:**
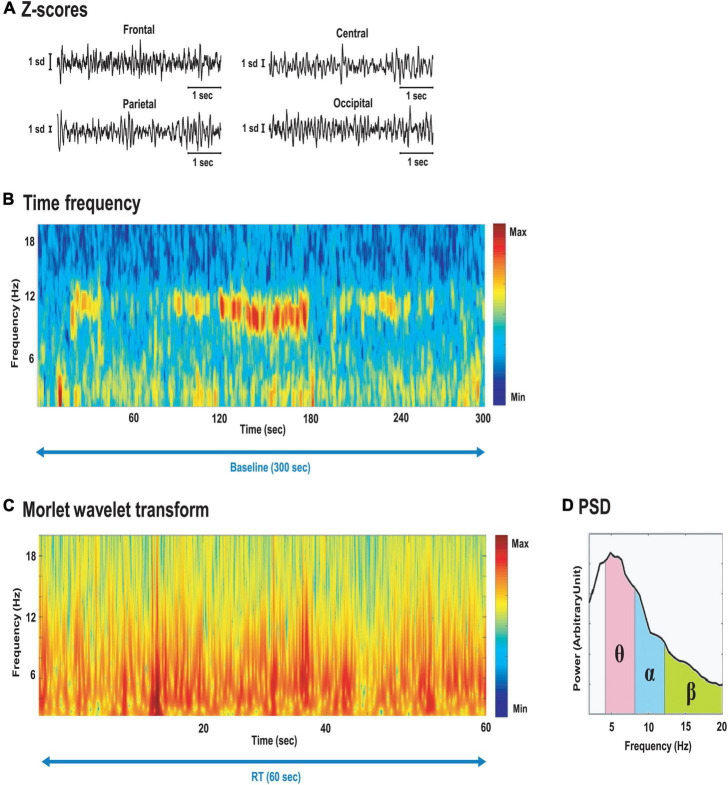
**(A)** LFP Z-scored trace for frontal, central, parietal and occipital electrodes; **(B)** Spectrogram of O2 electrode signal during the entire baseline parked (300 s). Note the emergence of alpha burst (8–12 Hz range) during the 1 min with eyes closed (from minute 2 to 3); **(C)** Time-resolved power spectral density (PSD, using complex Morlet wavelet transform); **(D)** resulting PSD.

All pre-processing and processing stages were performed with MATLAB (MathWorks ^®^, USA, 2016) built-in functions and the Chronux toolbox (Bokil et al., 2010). First, raw signals were pre-processed. Local field potentials (LFP) (Figure 3A) were (i) offline band-pass filtered between 0.1 and 100 Hz with a zero-phase shift filter function (zero-phase digital filtering *filtfilt* function) and (ii) detrended using local linear regression (*locdetrend* function from the Chronux toolbox: window-size 1 s, overlap 0.5 s) to remove baseline-drifting artifacts, and (iii) notch-filtered (*iirnotch* function), with the notch located at 50 Hz to remove possible line noise. Then, a band-pass filter with cut-off frequencies at 2–40 Hz was applied to remove muscle artifacts (Chen et al., 2010; Naqvi et al., 2015). The filtered EEG signals were then visually inspected to remove bad EEG channels based on low-quality acquisition. Signals were visualized using time-frequency representations (Figure 3B). Multitaper spectrogram method from the Chronux toolbox with time-bandwidth product of 2 and 4 slepian sequences of orthogonal data tapers was used to calculate power spectral density (PSD) of the LFP data with sliding windows of 2 and 0.2 s overlap.

Data analysis was performed using a transverse bipolar montage. Once correct EEG channels were identified, four pairs of electrodes were defined for the different cerebral regions (occipital, parietal, central, frontal). EEG signals were then normalized in order limit the influence of environmental changes, movements, and car vibrations, but also to limit intra- and inter-individual variability. For this, the LFP signal was expressed in z-score units. The z-score normalization used the mean and the standard deviation from each baseline (120 s) of each electrode: (i) baseline parked was used as reference for the static recovery period; (ii) baseline straight road was used as reference for the slalom period (comprising RT_start_ RT_++_ RT_stop_). Data recorded during baseline straight road were thus only considered for the normalization of slalom data and not analyzed thereafter.

Finally, instantaneous amplitude and phase from the LFP were obtained using a continuous Morlet wavelet transform (Figure 3C), with matcher filter construct parameters: Center frequency = 1 and bandwidth = 2, for the 2–40 Hz range. Due to their high flexibility, robust frequency information was extracted from the sum of these temporal windows to calculate PSD, reflecting the frequency content of each test period considered. For each of the four pairs of electrodes, PSD (Figure 3D) was averaged for each bandwidth of interest: theta (4–8 Hz), alpha (8–12 Hz), beta (12–26 Hz), and gamma (26–40 Hz) (Sauvet et al., 2014).

### Statistical Analysis

Two dependent variables were analyzed at the sample level: (i) Car-sickness ratings and (ii) frequency power of the EEG recordings. The evolution of each dependent variable was compared between parked baseline, slalom period, and recovery period via analysis of 5 periods (BasP, RT_start_, RT_++_, RT_stop_ and Recov). Car-sickness ratings were analyzed using a 5-level (BasP, RT_start_, RT_++_, RT_stop_ and Recov) repeated measures analysis of variance. EEG recordings were analyzed via 4 (pair of electrodes: Occipital, Parietal, Central, Frontal) × 5 (test period: BasP, RT_start_, RT_++_, RT_stop_ and Recov) repeated measures analysis of variance. As a prior for all the collected data, the condition of sphericity was also tested (Mauchly’s test). The *p*-value levels were corrected for possible deviations from sphericity by means of the Huynh–Feldt epsilon (ε). When significant differences were observed (*p* < 0.05), *post hoc* analysis was performed using a Fischer–Snedecor least significant difference test, allowing the results to be refined by comparing the modalities two by two. For each significant effect, the effect size was estimated using the partial eta squared (η*p*^2^). In addition, Spearman rank correlation analysis have been performed at the sample level between car-sickness ratings and power density from the four groups of frequencies for each cerebral area, according to the different test periods. All statistical analyses were performed using Statistica software ^®^ v.10 (Statsoft Inc., France). Data are presented as mean ± SEM for each assessment and significance levels as *p < 0.05, ^**^p < 0.01 and ^***^p < 0.001.

## Results

**FIGURE 4:**
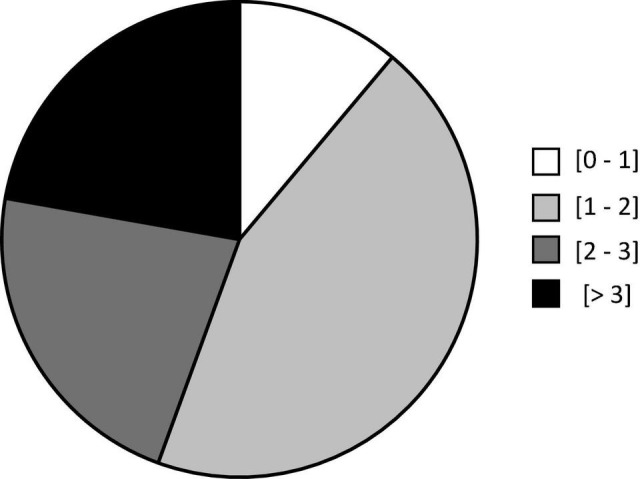
Distribution of maximum ratings reached by participants: white (0–1); light gray (1–2); dark gray (2–3) and black (>3).

All our participants were subjected to a maximum of 26 slaloms (i.e., 13 round trips) during the slalom period (19 ± 1 min) and reported at least some degree of car sickness. The distribution of participant ratings was as follows: maximum rating (0–1) 11.1%; (1–2) 44.4%; (2–3) 22.2%; (>3) 22.2% (Figure 4). Only one participant scored 4 in the last slalom. Globally, participants reached their maximum car-sickness ratings (1.6 ± 0.5) after 12.4 ± 2.9 min.

### Car-Sickness Ratings

**FIGURE 5:**
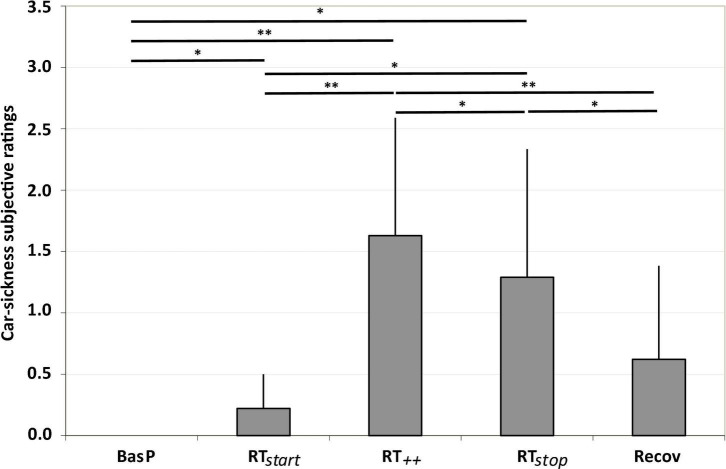
Car-sickness subjective ratings observed for each test period (BasP, RT_start_, RT_++_, RT_stop_ and Recov) (mean ± SEM; *n* = 9; *p < 0.05 and **p < 0.01 at risk α = 0.05).

A significant effect of the “test period” was observed on car-sickness ratings [*F*_(4, 32)_ = 15.94; ε = 0.49; *p* < 0.001; η*_p_*^2^ = 0.67]. As illustrated in Figure 5, although each participant began the experiment symptom-free, the ratings show that repeated low-frequency lateral movements rapidly induced symptoms. *Post hoc* analyses reveal a significant increase in all ratings during the slalom period compared to baseline. The ratings significantly increased from RT_start_ (+ 22% v. BasP; *p* < 0.05), reaching a maximum at RT_++_ (+ 163% vs. Baseline; *p* < 0.01). Then, the ratings decreased at the end of the slalom period (RT_stop_) (–13% vs. RT_++_; *p* < 0.05). Finally, stopping the slalom during the recovery period induced a significant decrease in the ratings (–38% vs. RT_++_; *p* < 0.01), which returned to baseline level.

### Electroencephalography Recordings

**FIGURE 6:**
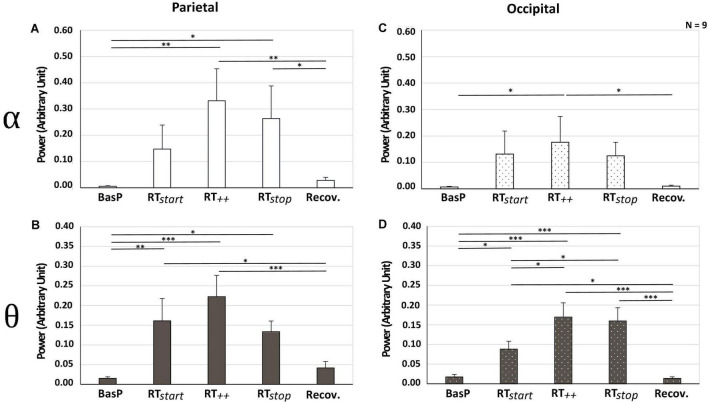
Power observed in alpha **(A–C)** and theta bands **(B–D)** for parietal **(A,B)** and occipital areas **(C,D)** for each test period (BasP, RT_start_, RT_++_, RT_stop_ and Recov) (mean ± SEM; *n* = 9; *p < 0.05, **p < 0.01, and ***p < 0.001 at risk α = 0.05).

As illustrated in Figure 6, power in alpha and theta bands increased in parietal and occipital areas during the slaloms compared with baseline. In the parietal area, a significant effect of “test period” was observed on alpha [*F*_(4, 32)_ = 4.16; ε = 0.49; *p* < 0.05; η*_p_*^2^ = 0.34] and theta power [*F*_(4, 32)_ = 6.42; ε = 0.74; *p* < 0.01; η*_p_*^2^ = 0.45] (Figures 6A,B, respectively). In comparison with baseline recordings, power values measured during the slaloms significantly increased. In alpha band, power significantly increased in RT_++_ (+ 32% vs. BasP; *p* < 0.01) and RT_stop_ (+ 26% vs. BasP; *p* < 0.01). In theta band, power also significantly increased in RT_start_ (+ 14% vs. Baseline; *p* < 0.01), RT_++_ (+ 20% vs. BasP; *p* < 0.001) and RT_stop_ (+ 12% vs. BasP; *p* < 0.05). Finally, for both frequency bands, the recovery period allowed all values to return to baseline level, with a significant decrease in alpha (–23% vs. RT_++_; *p* < 0.05) and theta bands (–12% vs. RT_++_; *p* < 0.001 and –8% vs. RT_stop;_ *p* < 0.001). In the occipital area, a significant effect of “test period” was observed on alpha [*F*_(4, 32)_ = 2.68; ε = 0.27; *p* < 0.05; η*_p_*^2^ = 0.25] and theta power [*F*_(4, 32)_ = 6.42; ε = 0.74; *p* < 0.01; η*_p_*^2^ = 0.45] (Figures 6C,D, respectively), with power increasing during slaloms in both frequency bands. In alpha band, power reached maximum level in RT_++_, which was significantly higher than baseline (+ 17% vs. BasP; *p* < 0.05). In theta band, power also significantly increased in comparison with baseline in RT_start_ (+ 7% vs. Baseline; *p* < 0.05), RT_++_ (+ 15% vs. BasP; *p* < 0.001) and RT_stop_ (+ 14% vs. BasP; *p* < 0.001). Theta power continued to increase from RT_start_, reaching maximum levels in RT_++_ (*p* < 0.05) and RT_stop_ (*p* < 0.05). Finally, all values returned to baseline levels during the recovery period, with a significant decrease in alpha (–14% vs. RT_++_; *p* < 0.05) and theta bands (–7% vs. RT_start_; *p* < 0.05; –13% vs. RT_++_; *p* < 0.001 and –13% vs. RT_stop;_ *p* < 0.001). No other significant difference was observed regarding the other bandwidths (beta, gamma), nor the other cerebral areas (central, frontal).

### Relationship Between Car-Sickness Ratings and Electroencephalography Responses

The mean power for the four groups of frequencies was calculated for each cerebral areas according to the different test periods. As so, it was possible to determine the average effect of increasing car-sickness ratings on EEG responses at the sample level by using linear regressions. Results are shown in Supplementary Figure 1.

For the frontal and occipital areas, the increase in car-sickness ratings was significantly correlated with the increase in gamma power. In contrast, no significant correlation was observed for the central area, regardless the frequency range. However, for the parietal area, there was a linear increase of the mean power in alpha and beta rhythm with car-sickness ratings.

## Discussion

This study is the first to evaluate the impact of 0.2 Hz lateral movements in real driving conditions on both the occurrence of car-sickness symptoms and associated modifications in cerebral activity. Our findings from car-sickness ratings confirm that these very low-frequency lateral movements induce car-sickness symptoms. Furthermore and remarkably, our results show several changes in cerebral activity appearing when participants were exposed to these movements. Both theta and alpha power in the parietal and occipital areas increased with car-sickness ratings. When the 0.2 Hz lateral movements stopped, spectral power in these low-frequency bands returned to baseline levels in both cerebral areas concomitantly with decreased car-sickness ratings.

### Car-Sickness Ratings

There was a significant increase in car-sickness ratings from RT_start_, reaching a maximum level during RT_++_ usually about half-way through the slalom period. This suggests a cumulative effect of the slaloms on participants’ subjective ratings, in agreement with findings from previous studies under real driving conditions using similar methodology (time of exposure, successive 0.2 Hz lateral movements in slaloms linked by U-turns, likert-type scale; Wada et al., 2012; Wada and Yoshida, 2016; Kuiper et al., 2018), where the evolution of car-sickness ratings was comparable. A cumulative increase in sickness ratings was also reported in dynamic simulator conditions (continuous lateral slalom; Lin et al., 2007, 2013; Chen et al., 2010; Chuang et al., 2016). Our results thus confirm that progressive exposure to lateral movements at 0.2 Hz induces car sickness, as already demonstrated for vertical movements in laboratory conditions.

Interestingly, the last round-trip ratings were significantly lower than the higher ratings, indicating a habituation effect from exposure to regular movements (slaloms). These results differ from previous findings indicating either continuous increase (Lin et al., 2007; Chen et al., 2010; Kuiper et al., 2018) or stagnation of car-sickness ratings (Wada and Yoshida, 2016). Several parameters such as the length of the slalom, the presence of a U-turn between slaloms, and the instructions given regarding head positioning no doubt contribute to these discrepancies in results. First, in real driving conditions, Kuiper et al. (2018) reported a continuous increase in sickness ratings from the beginning of the test to the end. They used a long slalom (520 m, i.e., twice the length of our slalom) with a tight U-turn before returning along the same line of pylons. While no information is available on the speed at which the U-turn was negotiated, it can be assumed that the lateral acceleration in this configuration was not negligible, and that the vehicle still followed a curved path to negotiate the following slalom. Other studies in simulated conditions using continuous slaloms (without U-turns) did not report any stagnation or decrease in sickness ratings either (Lin et al., 2007; Chen et al., 2010). It can thus be hypothesized that car-sickness ratings will continue to increase if there are no breaks in lateral acceleration between slaloms (U-turns). Second, using a method that was quite similar to ours in terms of movement frequency (0.2 Hz lateral movements) and track (2 different lines of 150 m lateral slaloms with U-turns negotiated at a reduced speed), Wada and Yoshida (2016) showed a stagnation of car-sickness ratings. This suggests either that participants were beginning to experience a habituation effect, or that they had simply reached their maximum discomfort level. Nonetheless, although the level of acceleration during the slaloms in their study resembled ours, their U-turns had different radii of curvature (6 m and 10 m), which could impact the level of lateral acceleration. Golding (2006) stated that repeated exposure to noxious movements using several blocks is required for a habituation process. The configuration of their track, 2 lines of pylons separated by a large U-turn, promotes such block perception, although lateral acceleration during the U-turns needs to be significantly reduced (as in the 10 m radii condition) to initiate this process. Third, another major difference between our study and Wada et al. (2012) and Kuiper et al. (2018) is that their procedures controlled head position. In Wada et al. (2012), participants had to move their head from one side to another (centrifugal vs. centripetal side depending on the tested condition), while in Kuiper et al. (2018), participants watched a screen fixed in front of them on the dashboard. In our study, head positioning was not imposed. Thus, it may be that the break allowed by the large U-turns between repeated slaloms, coupled with free head positioning, led to participants becoming accustomed to the lateral movements (habituation process).

Finally, stopping the slalom induced a significant decrease in symptoms after the recovery period (5 min), with a return to baseline levels. Unfortunately, comparisons with previous studies remain limited, as few addressed this specific period and almost none reported its effect on car-sickness ratings. To the best of our knowledge, only Wada et al. (2012), Wada and Yoshida (2016) indicated that the longer the recovery period after the slalom period (5 or 10 min), the less severe the car sickness.

### Electroencephalography Recordings

Importantly in these real driving conditions, EEG recordings showed that theta and alpha powers changed significantly in the parietal and occipital areas during the slalom period. While previous work on cerebral changes occurring with motion sickness also reported changes in these specific power bands and cerebral areas (Wu, 1992; Wood et al., 1994; Kim et al., 2005; Lin et al., 2007, 2013; Chen et al., 2010; Naqvi et al., 2015), their findings varied from increase to decrease, as well as stagnation. Discrepancies in methodology (rotating chair, parallel swing, oscillatory picture, VIMS, dynamic driving simulator), materials (EEG headsets with 16, 32, or 128 electrodes), or experimental tasks (activity, passenger vs. driver, exploration task, etc.) may explain the widely varying results. Moreover, although rarely acknowledged in previous studies, symptoms and physiological changes may vary according to type of paradigm/set-up. Experiments with no platform movement may be intended to address cyber sickness (oscillatory picture, virtual reality, VIMS) (Kim et al., 2005; Naqvi et al., 2015; Liu et al., 2017), those with platform movement to address motion sickness (parallel swing, rotating chair) (Wu, 1992; Wood et al., 1994; Hu et al., 1999), and those using a car to address car sickness (Wada et al., 2012; Wada and Yoshida, 2016). The contrasting findings on the evolution of theta and alpha power (increase vs. decrease) may depend on the presence/absence of platform movements. Protocols including platform movements and addressing motion sickness were shown to induce an increase in these power bands (Wu, 1992; Wood et al., 1994). In contrast, those with no platform movement and that relied only on visual stimulations to address cyber sickness reported a decrease in theta and alpha power (Kim et al., 2005; Naqvi et al., 2015; Liu et al., 2017). However, protocols involving a dynamic driving simulator using platform movements and a virtual environment (VR) that provided visual, vestibular, and proprioceptive solicitations (Lin et al., 2007, 2013; Chen et al., 2010; Chuang et al., 2016) showed an increase in theta and alpha power in occipital and parietal areas (Lin et al., 2007, 2013; Chen et al., 2010). The particular type of sickness induced by these protocols—cyber sickness, motion sickness or a combination of both—remains somewhat difficult to identify.

Our results indicate an increase in theta power in occipital and parietal areas from RT_start_, simultaneously with an increase in car-sickness ratings. Several studies reported that theta power increases during tasks requiring specific sensory attention (e.g., focus on visual and/or auditory stimuli), particularly in areas dedicated to sensory integration, suggesting that theta oscillations are associated with sensory attention (Keller et al., 2017; Wang et al., 2018). In our study, sensory inputs stimulated by 0.2 Hz lateral movements were not only visual, from participants’ watching the slaloms progress, but also vestibular and proprioceptive, as induced by the movements of the car and their own bodies. In addition, since participants in our study had never previously been exposed to such stimuli, the increased theta power in occipital and parietal areas from the first slalom on may have arisen from their attention being focused on integrating these new sensory inputs. Theta oscillations are believed be involved in the synchronization of sensory inputs between several cerebral areas (Caplan et al., 2003; Raghavachari et al., 2006; Chen et al., 2010). As such, theta oscillations may not only help focus attention on unusual sensory inputs, but also help optimize multisensory integration through the synchronization of the different cerebral areas involved.

During RT_++_, where car-sickness ratings were highest, increased alpha power in occipital and parietal areas was observed. Recent works identified alpha oscillations not only as markers of sensory input mitigation when present in a specific cortical area (Keller et al., 2017), but also as a local marker of cortical excitability level, high alpha power being associated with low cortical activity (Lebar et al., 2017). In our study, the increase in alpha power may reflect reduced integration of sensory inputs (vestibular, proprioceptive, and visual) in order to minimize their impact (sensory conflict) and/or reduced excitability of occipital and parietal areas. According to the sensory gating model, an automatic inhibitory function may be related to human higher cognitive processing (Gallicchio and Ring, 2019; Mabuchi et al., 2020). More precisely, the function of alpha is to exert inhibitory control across the cerebral cortex, whereby higher alpha indicates stronger neuronal inhibition and lower alpha indicates greater release from inhibition (Klimesch et al., 2007; Klimesch, 2012). Beyond a simple drop in sensory integration, some authors even suggested that the presence of alpha power in the somatosensory areas may reflect the suppression of vestibular inputs to eliminate the conflict with participants’ visual perception in driving dynamic simulators (Chen et al., 2010; Lin et al., 2013). In addition, alpha power can also be interpreted as a marker of relaxation or drowsiness (Sauvet et al., 2014). Drowsiness is currently considered one symptom of motion sickness (Lackner, 2014). Therefore, alpha power in occipital and parietal areas might reflect the participants’ need for sleep to limit their discomfort. However, it should be remembered that closing one’s eyes alone will not lower the discomfort level, as it only blocks visual information. As highlighted by Wada and Yoshida’s (2016) study on car-sickness symptoms occurring with eyes closed vs. eyes open, closing one’s eyes intensifies symptoms due to conflict between perceived sensory inputs (visual: no movement felt; proprioceptive/vestibular: movements felt) (Schmäl, 2013; Koohestani et al., 2019). The increase in theta power reached a peak during RT_++_. Several studies reported that theta power increases during learning phases and/or in sensory information memorization processes (Herrmann et al., 2016), and the increase in theta power observed here may play a role in such processes related to noxious movement perception.

During RT_stop_, the increase in alpha oscillations was no longer observed in the occipital area. Thus, alpha oscillations only appeared to produce a boost effect in RT_++_, suggesting a sensory mitigation of visual inputs when symptoms reached their highest level. In contrast, the increase previously observed in the parietal area during RT_++_ lasted until RT_stop_. This supports the idea that a longer increase in alpha oscillations is required in the parietal area to reduce sensory input integration (proprioceptive and vestibular; Keller et al., 2017). Theta power remained at a higher level in both occipital and parietal areas during RT_stop_, indicating that the sensory input memorization processes might still be activated. Several studies reported that repeated exposure to noxious movements can trigger habituation (Kim et al., 2005; Golding, 2006). In our study, participants had already experienced several slaloms when the last round trip occurred, but their car-sickness ratings decreased. One possible explanation is that the continuous increase in theta power may reflect habituation and desensitization to this kind of movement (0.2 Hz lateral movements). This suggests that such cerebral changes may help limit the persistence of car-sickness symptoms.

Once the movements stopped during recovery, an adaptation of cerebral activity was observed: theta and alpha power returned to baseline levels concomitantly with a decrease in car-sickness ratings. Thus, the cerebral processes activated during the slalom period (RT_start_, RT_++_, RT_stop_) to limit car-sickness symptoms appear no longer to have been required. While it would be interesting to compare these results with findings in the literature, no study has previously focused on recovery and its associated changes in cerebral activity.

This study performed a preliminary analysis of EEG signals to determine overall changes in cerebral activity based on a bipolar montage. Several signals located in central and frontal areas were rejected for analysis after visual inspection, which may have limited the observation of significant differences in these areas. More advanced EEG analyses (ICA, time/frequency analysis, correlation/regression) would be required to determine the exact position and the kinetics of the sources of these cerebral changes. Moreover, since we observed large inter-individual differences in sensitivity to car sickness and in cerebral activity changes, it would be interesting to examine a larger sample to better understand these discrepancies, as well as for categorization purposes. Despite these limitations, our innovative study allowed car-sickness levels and cerebral activity to be measured in real driving conditions. Further research should explore the full kinetics of the fluctuations observed in car-sickness levels in relation to cerebral activity.

## Conclusion

This study revealed for the first time in real driving conditions that car-sickness symptoms appear concomitantly with changes in EEG activity, mainly in the cerebral areas involved in sensory integration. In particular, we found theta and alpha power in parietal and occipital areas to increase with increasing severity of car sickness. These findings are consistent with previous non-driving studies conducted using platform movements in laboratory conditions, confirming that theta and alpha power are sensitive indicators of car sickness. Every participant was sensitive to the car movements in our study (0.2 Hz lateral movements) and suffered some degree of car sickness. Hence, these specific movements should be avoided, especially in the currently emerging autonomous vehicles, to improve comfort and well-being on board.

## Data Availability Statement

The raw data supporting the conclusions of this article could be obtained by asking the corresponding author.

## Ethics Statement

The studies involving human participants were reviewed and approved by the Local Ethics Committee of Aix-Marseille University in accordance with the ethical standards laid down in the 1964 Declaration of Helsinki. The patients/participants provided their written informed consent to participate in this study.

## Author Contributions

EH and ClB performed the experiment and the measurements. EH analyzed the data and wrote the manuscript. LB, ClB, and ChB reviewed the manuscript. All authors have contributed equally to the design of the experiment.

## Conflict of Interest

The authors declare that the research was conducted in the absence of any commercial or financial relationships that could be construed as a potential conflict of interest.

## Publisher’s Note

All claims expressed in this article are solely those of the authors and do not necessarily represent those of their affiliated organizations, or those of the publisher, the editors and the reviewers. Any product that may be evaluated in this article, or claim that may be made by its manufacturer, is not guaranteed or endorsed by the publisher.

## Supplementary Material

Supplementary Figure 1 Illustration of the mean frequency power at increasing levels of motion sickness. The linear regression from the Spearman rank correlation analysis at the sample level are shown for the 4 frequency ranges: theta (4–8 Hz), alpha (8–12 Hz), beta (12–26 Hz), and gamma (26–40 Hz). *R*^2^ are provided for the different linear regressions.

## Abbreviations

BasP: baseline parked
BasS: baseline straight road
RT_start_: the first round trip
RT_++_: the highest-rated round trip
RT_stop_: the last round trip
Recov: recovery.
